# Comparative effectiveness of budesonide/formoterol combination and fluticasone/salmeterol combination among chronic obstructive pulmonary disease patients new to controller treatment: a US administrative claims database study

**DOI:** 10.1186/s12931-015-0210-x

**Published:** 2015-04-23

**Authors:** David M Kern, Jill Davis, Setareh A Williams, Ozgur Tunceli, Bingcao Wu, Sally Hollis, Charlie Strange, Frank Trudo

**Affiliations:** HealthCore, Inc., 123 Justison St, Suite 200, Wilmington, DE 19801-5134 USA; AstraZeneca Pharmaceuticals, 1800 Concord Pike, Wilmington, DE 19850 USA; AstraZeneca Pharmaceuticals, One MedImmune Way, Gaithersburg, MD 20878 USA; AstraZeneca Pharmaceuticals, Alderley Park, Cheshire, UK; Department of Medicine, Division of Pulmonary and Critical Care Medicine, Medical University of South Carolina, Charleston, SC 29425 USA

**Keywords:** Chronic obstructive pulmonary disease (COPD), Inhaled corticosteroid/long-acting β_2_-agonist combinations (ICS/LABA), Comparative effectiveness, Controller treatments, Administrative claims

## Abstract

**Background:**

Inhaled corticosteroid/long-acting β_2_-agonist combinations (ICS/LABA) have emerged as first line therapies for chronic obstructive pulmonary disease (COPD) patients with exacerbation history. No randomized clinical trial has compared exacerbation rates among COPD patients receiving budesonide/formoterol combination (BFC) and fluticasone/salmeterol combination (FSC) to date, and only limited comparative data are available. This study compared the real-world effectiveness of approved BFC and FSC treatments among matched cohorts of COPD patients in a large US managed care setting.

**Methods:**

COPD patients (≥40 years) naive to ICS/LABA who initiated BFC or FSC treatments between 03/01/2009-03/31/2012 were identified in a geographically diverse US managed care database and followed for 12 months; index date was defined as first prescription fill date. Patients with a cancer diagnosis or chronic (≥180 days) oral corticosteroid (OCS) use within 12 months prior to index were excluded. Patients were matched 1-to-1 on demographic and pre-initiation clinical characteristics using propensity scores from a random forest model. The primary efficacy outcome was COPD exacerbation rate, and secondary efficacy outcomes included exacerbation rates by event type and healthcare resource utilization. Pneumonia objectives included rates of any diagnosis of pneumonia and pneumonia-related healthcare resource utilization.

**Results:**

Matching of the identified 3,788 BFC and 6,439 FSC patients resulted in 3,697 patients in each group. Matched patients were well balanced on age (mean = 64 years), gender (BFC: 52% female; FSC: 54%), prior COPD-related medication use, healthcare utilization, and comorbid conditions. During follow-up, no significant difference was seen between BFC and FSC patients for number of COPD-related exacerbations overall (rate ratio [RR] = 1.02, 95% CI = [0.96,1.09], *p* = 0.56) or by event type: COPD-related hospitalizations (RR = 0.96), COPD-related ED visits (RR = 1.11), and COPD-related office/outpatient visits with OCS and/or antibiotic use (RR = 1.01). The proportion of patients diagnosed with pneumonia during the post-index period was similar for patients in each group (BFC = 17.3%, FSC = 19.0%, odds ratio = 0.92 [0.81,1.04], *p* = 0.19), and no difference was detected for pneumonia-related healthcare utilization by place of service.

**Conclusion:**

This study demonstrated no difference in COPD-related exacerbations or pneumonia events between BFC and FSC treatment groups for patients new to ICS/LABA treatment in a real-world setting.

**Trial registration:**

ClinicalTrials.gov identifier NCT01921127.

## Background

It is estimated that over 12 million Americans have been diagnosed with chronic obstructive pulmonary disease (COPD) [1]. This disease is characterized by poor and worsening lung function, and is associated with immense patient and societal burdens [2]. In recognition of this enormous negative impact, [3,4] the Global Initiative for Chronic Obstructive Lung Disease (GOLD) identified the prevention and treatment of exacerbations as leading priorities for the management of COPD [5].

The GOLD 2014 treatment guidelines support the initiation of controller medications for patients with COPD symptoms and exacerbations [5]. Inhaled corticosteroid/long-acting β_2_-agonist combinations (ICS/LABA) are endorsed by the GOLD guidelines as a first-line maintenance therapy for COPD patients with a history of exacerbations. Currently, both budesonide/formoterol combination (BFC, 160/4.5 μg) [6] and fluticasone/salmeterol combination (FSC, 250/50 μg) [7] are indicated for maintenance treatment of airflow obstruction in COPD patients in the United States (US).

As of now, no randomized clinical studies have compared the treatment effectiveness of FSC versus BFC in COPD. Limited indirect comparison from RCT is available; however, given the various definitions of outcomes as well as variability in disease severities included, comparative effectiveness remains inconclusive [8]. With limited comparative data available in the US, at least one structured literature review delineated the distinctive challenges associated with conducting comparative research into BFC and FSC therapies for COPD [9,10]. One US-based retrospective study, which evaluated sizeable matched cohorts of COPD patients for 3–6 months, showed that BFC and FSC treatments had comparable effectiveness with respect to exacerbation rates and pneumonia events; however, BFC patients required less rescue medication during the observation period, which could be an indication that their symptoms were controlled better [11]. A 12-month, population based retrospective study in Canada with matched COPD cohorts reported that BFC-treated patients were associated with lower likelihood of emergency department (ED) visits, inpatient hospitalizations and anticholinergic medication use versus FSC-treated patients during the follow up period [12]. Similarly, a study that evaluated matched cohorts queried from primary care records linked with Swedish hospital, pharmaceutical and cause of death registries, reported that long-term BFC treatment was associated with fewer exacerbations, based on healthcare utilization measures, compared with FSC therapy [13]. The same Swedish study also found lower rates of pneumonia and pneumonia related death in patients treated with BFC compared to those treated with FSC [14].

The primary objective of this study was to compare the real-world effectiveness of BFC and FSC treatments among COPD patients new to ICS/LABA combination therapy in a population of US managed care enrollees. One important goal was to help address the evidence gap of specific relevance to the US, given previous studies were mostly carried out outside of the US where ICS/LABA formulation and delivery device variations are different than in the US. This study also compared pneumonia rates for patients initiating either BFC or FSC as pneumonia is an important safety endpoint among patients receiving ICS/LABA combination therapy [5,15,16].

## Methods

### Study design and population

Patients initiating BFC (160/4.5 μg) or FSC (250/50 μg) between 03/01/2009 and 03/31/2012 were identified in the HealthCore Integrated Research Environment (HIRE) and followed for 12 months in this retrospective cohort study. The index date was defined as the first prescription fill for BFC or FSC during the intake period. At the time of this study, the HIRE contained longitudinal claims data of more than 31 million enrollees from all US census regions. Handling of study materials complied with applicable Health Insurance Portability and Accountability Act (HIPAA) rules. Data were from a limited dataset with de-identified patient information to preserve patient confidentiality. This non-experimental study was conducted under the Research Exception provisions of the Privacy Rule, 45 CFR 164.514(e), and was exempt from Investigational Review Board (IRB) approval, removing the need for individual patient authorization.

### Inclusion/exclusion criteria

**Figure 1:**
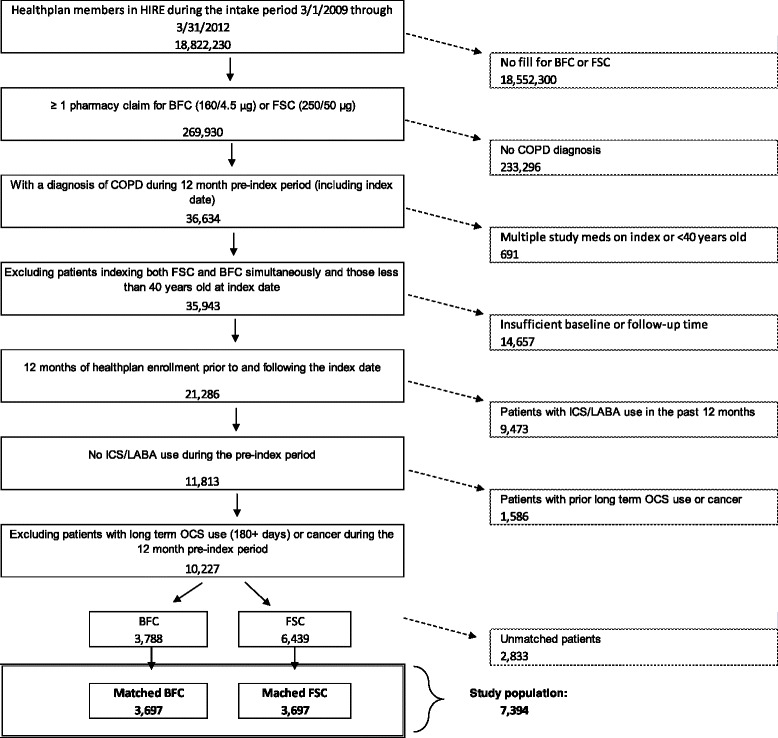
Patient selection.

Patients were required to be naïve to ICS/LABA therapy one year prior to initiating BFC (160/4.5 μg) or FSC (250/50 μg), where the pharmacy claim date of treatment initiation during the intake period was considered the index date. Patients were required to be at least 40 years old at the index date, and have at least 12 months of continuous health plan enrollment, including medical and pharmacy eligibility, prior to (pre-index period) and following the index date (post-index period). Diagnosis criteria required for inclusion were at least one inpatient visit with a primary diagnosis for COPD (ICD-9-CM diagnosis code 491.xx, 492.xx, or 496.xx), and/or, at least, one ED visit with a COPD diagnosis (either primary or secondary), and/or, at least, two other medical claims with a COPD diagnosis (either primary or secondary) during the pre-index period. Patients diagnosed with cancer and those who received ≥180 days of oral corticosteroid (OCS) therapy during the 12-month pre-index period, and those initiating both study medications on the same date were excluded from the study. The number of eligible individuals remaining after each inclusion criterion is illustrated in Figure 1.

### Measurements

#### Primary outcome

The primary outcome measure was the rate of COPD exacerbations, calculated as the number of exacerbations during the 12-month post-index period. A COPD exacerbation was defined as a COPD-related inpatient hospitalization (inpatient hospitalization with a primary diagnosis for COPD), and/or a COPD-related ED visit (an ED visit with a diagnosis for COPD in any position) and/or a pharmacy claim for OCS and/or antibiotics on the same day as or within 10 days after an office/outpatient visit with a diagnosis for COPD. Exacerbations occurring within 14 days of each other were considered a single event. ED visits resulting in a hospital stay were counted as an inpatient hospitalization only. Any OCS or antibiotic prescription fill occurring within 14 days of a COPD-related ED/inpatient hospitalization was counted as the ED/hospitalization only. Multiple OCS and/or antibiotic fills within 10 days of the same outpatient visit were only counted as one event.

### Sensitivity analysis of primary outcome

#### Stopping follow-up after switching ICS/LABA therapy

Follow-up was stopped at the date a patient filled an ICS/LABA that was different from their index medication. Analysis only included exacerbation events and follow-up time that occurred prior to the treatment modification during the 12-month post-index period. Patients who did not switch ICS/LABA therapy were followed for the entire 12 months.

#### Using all available follow-up time

Analysis included all available follow-up, for which patients could be followed beyond 12 months. Follow-up ended at the end of health plan enrollment or the end of the study period.

#### Severe COPD exacerbation events

Analysis only included events associated with COPD-related inpatient hospitalizations and COPD-related ED visits.

### Secondary outcomes

1. ***Secondary outcome of exacerbation: time to first event and exacerbation event types.*** The time to the first COPD exacerbation was evaluated to mitigate the effect of potential switching or discontinuation of the index ICS/LABA medication due to treatment failure. The exacerbation rates for each type of exacerbation type (i.e., COPD-related inpatient hospitalization, COPD-related ED visit, and pharmacy claim for OCS and/or antibiotics with a COPD-related office/outpatient visit) were analyzed separately.
2. ***Healthcare utilization (COPD-related and all-cause) and COPD-related medication use.*** Included in this measure were the number of visits by place of service (inpatient and ICU stays, ED, outpatient and skilled nursing facility). COPD-related utilization was defined as the presence of a COPD diagnosis code (primary diagnosis for inpatient stays or any diagnosis for other places of service) for that visit. All-cause utilization included healthcare use for any reason or diagnosis. For COPD-related medication use, the proportion of patients with at least one prescription claim for ICS, LABA, long-acting muscarinic antagonist (LAMA), short acting β2-agonist (SABA), short-acting muscarinic antagonist (SAMA), SABA/SAMA combination, OCS, leukotriene receptor antagonist (LTRA), roflumilast, theophylline, omalizumab and antibiotics were identified.
3. ***Medication adherence*.** Adherence was measured as the proportion of days covered (PDC) with the index medication. PDC was calculated as the days supply of the index medication during the post-index period divided by the post-index period (365 days) [17].
4. ***Pneumonia events.*** The diagnosis criteria for identifying viral, bacterial and aspiration pneumonia in the administrative claims database was validated using medical chart review as the referent standard, and was found to have a positive predictive value of 80% and used for the following analyses.
  The outcome of pneumonia (defined as having a claim with a primary or secondary diagnosis with the following diagnosis codes: ICD-9-CM 480.xx – 486.xx) was examined in three different ways between the two cohorts. First, the pneumonia rate within each cohort was calculated as the proportion of COPD patients with at least one pneumonia diagnosis during the 12 month post-index follow-up. Second, the time to first pneumonia diagnosis was analyzed to account for possible differences in timing of the diagnoses found in the first analysis. Lastly, pneumonia-related utilization was examined by each place of service: inpatient, ED, and outpatient visit.

### Data analyses

The retrospective study design allowed for the inclusion of all patients who met all inclusion/exclusion criteria and were successfully matched on propensity scores. Based on an expected exacerbation rate for an ICS/LABA patient of 0.5 per person/year, [12] a rate reduction for a BFC patient of 20%, alpha level of 0.05, 90% power, and dispersion effect of 1.2, power calculations resulted in a minimum necessary sample size of 1,457 matched subjects in each cohort.

To reduce selection bias and create more comparable BFC and FSC cohorts, propensity score matching was used to adjust for confounders measured pre-index [18-20]. The propensity score for each individual was estimated as the probability of receiving BFC, conditional on observed baseline characteristics. Random forests were used to estimate the propensity scores as the probability of receiving BFC therapy [21,22]. The outcome variable in the model was dichotomous, indicating whether a patient received BFC (1) or FSC (0) therapy. Treatment cohorts were considered well balanced when the p-value for the difference between groups was p > 0.05 for each of the pre-specified variables listed below.

It was specified a priori that study samples would be balanced on age, gender, prior asthma diagnosis, and pre-index characteristics including COPD-related inpatient hospitalizations and ED visits, OCS fills, antibiotic fills, SABA and/or SABA/SAMA fills, LABA fills, and LAMA fills. Although optional, balance was also achieved for an additional set of variables, including pre-index exacerbations and comorbid conditions, among others. All statistical models included covariates identified during the 12 month pre-index period as not being balanced after matching and also the analogous pre-index variable; for example, when analyzing the number of COPD-related hospitalizations in the post-index, the model also controlled for the number of pre-index COPD-related hospitalizations.

A generalized linear model (GLM) from a negative binomial distribution with a log link function was used to model the primary outcome (including all sensitivity analyses) of the number of exacerbations. Covariate adjusted rates, rate ratios, 95% CIs, and *p*-values are reported. The time to first COPD exacerbation and time to first pneumonia diagnosis were analyzed using Cox proportional hazards models. Hazards ratios, 95% CIs, and *p*-values were reported alongside Kaplan-Meier curves of the time to first event. Healthcare utilization was analyzed via negative binomial regression models for the number of events during the post-index period, normal linear regression was used for testing differences in the proportion of days covered with index medication, ordinal logistic regression was used to test for differences in the distribution of number of fills for the index medication (1, 2, 3, or 4 or more), and logistic regression models were used for the outcomes of at least one event versus zero events, including the outcome of pneumonia.

Intent-to-treat populations were used for all analyses. All outcomes and statistical analysis methods were defined a priori in the study protocol, and no adjustment was made for multiple testing. Propensity scores were obtained using R, [23] while all other analysis was performed using SAS version 9.4 (Cary, NC).

## Results

### Cohort matching

A total of 3,788 BFC and 6,439 FSC initiators met all inclusion/exclusion criteria prior to matching. Table 1 presents the balance of demographics and pre-index variables before and after matching. Following propensity score matching, a total of 3,697 patients from each cohort were matched. All pre-specified variables were well balanced in addition to most of the optional variables, including measures of prior COPD exacerbations and diagnosis of pneumonia. Variables not pre-specified to be matched, and remaining imbalanced after the matching algorithm was complete, were the sum of inpatient hospital stays >5 days (0 vs. ≥ 1), pre-index LTRA use (0, 1, ≥2), geographic region (northeast, midwest, south, or west), peripheral vascular disease/atherosclerosis (yes vs. no), and index prescribing physician specialty (pulmonologist, internal medicine, family medicine/general practitioner, cardiologist, allergist/immunologist, non-physician, or other specialty). These variables were included as covariates in all statistical models.

**Table 1:** **Propensity score matching results – Required matched variables and exacerbation-related variables**

**Matching criteria**	**Prior to matching**					**After matching**				
	**BFC (n = 3788)**		**FSC (n = 6439)**		**P-value**	**BFC (n = 3697)**		**FSC (n = 3697)**		**P-value**
**Priority matching**										
**Demographics**										
Age (mean, SD)	63.7	11.4	65.0	12.0	<0.0001	63.7	11.5	64.0	11.8	0.3139
Female (n, %)	1981	52.3%	3535	54.9%	0.0108	1932	52.3%	1989	53.8%	0.1841
Prior asthma diagnosis (n, %)	1371	36.2%	2078	32.3%	<0.0001	1320	35.7%	1280	34.6%	0.3299
**COPD severity**										
Prior COPD inpatient visits (mean, SD)	0.13	0.40	0.15	0.42	0.0252	0.13	0.40	0.14	0.43	0.5613
Prior COPD ED visits (mean, SD)	0.18	0.52	0.18	0.52	0.6520	0.18	0.52	0.19	0.55	0.3530
Prior OCS fills (mean, SD)	1.26	1.74	1.01	1.39	<0.0001	1.22	1.68	1.17	1.54	0.1823
Prior antibiotic fills (mean, SD)	2.74	2.75	2.48	2.61	<0.0001	2.70	2.72	2.70	2.81	0.9441
**COPD medications**										
Prior SABA or SABA/SAMA fills (mean, SD)	2.99	4.45	2.35	3.83	<0.0001	2.89	4.37	2.88	4.41	0.9161
Prior LABA fills (mean, SD)	0.24	1.35	0.11	0.92	<0.0001	0.22	1.29	0.16	1.10	0.1169
Prior LAMA fills (mean, SD)	1.19	2.61	0.98	2.32	<0.0001	1.15	2.55	1.11	2.53	0.5224
**Optional matching**										
Pre-index exacerbations (mean, SD)	1.08	1.25	0.94	1.14	<0.0001	1.07	1.24	1.05	1.23	0.5276
Pre-index exacerbations, ≥1 (n, %)	2337	61.7%	3795	58.9%	0.0060	2267	61.3%	2282	61.7%	0.7199
due to COPD related inpatient visit	426	11.2%	863	13.4%	0.0015	420	11.4%	447	12.1%	0.3291
due to COPD related ED visit	536	14.1%	938	14.6%	0.5615	523	14.1%	553	15.0%	0.3225
due to COPD outpatient/office visit + OCS and/or antibiotics	1797	47.4%	2575	40.0%	<0.0001	1732	46.8%	1663	45.0%	0.1073
Pneumonia diagnosis (n, %)	849	22.4%	1567	24.3%	0.0270	823	22.3%	867	23.5%	0.2230

Additional balance was achieved on: index month; prescribing physician a pulmonologist; hospitalization due to cardiovascular disease, hospitalization due to pneumonia; hospitalization due to asthma; long term oxygen use; comorbid conditions (pulmonary hypertension, chronic respiratory failure, anxiety, depression or psychotropic drug use, coronary artery disease, left ventricle failure, diabetes, congestive heart failure, hypertension, and stroke); prior medication use categorized as 0, 1, or 2+ fills for the following classes: OCS, antibiotics, ICS, LAMA, LABA, roflumilast, theophylline, SABA, SAMA, SABA/SAMA combination, omalizumab, and any cardiovascular related); influenza vaccination; pneumococcal vaccination.

### Demographics and baseline characteristics

Patients were 64 years old on average in each treatment group and a similar proportion of patients in each cohort were female (52% of BFC and 54% of FSC initiators) (Table 1). Additionally, patients had similar rates of previously diagnosed pneumonia (22% BFC, 23% FSC), asthma (36% BFC, 35% FSC), and hypertension (69% BFC, 68% FSC), among other conditions, during the 12-month pre-index period. Use of prior respiratory medications was also similar, including fills of OCS, ICS, LAMA, SABA, and SABA/SAMA combination.

### Primary outcome

For the primary outcome, the covariate adjusted rate of COPD exacerbations was no different for patients initiating BFC (exacerbation rate = 0.88) or FSC (exacerbation rate = 0.86) during the 12 months following the initiation of therapy (rate ratio [RR] = 1.02, 95% CI = [0.96, 1.09], *p* = 0.56), as shown in Table 2. There were 48% of BFC patients and 47% of FSC patients experiencing at least one COPD exacerbation during the follow-up period. There was also no difference in the exacerbation rate for each of the COPD exacerbation event types between the two groups. Patients in the BFC and FSC cohorts had similar rates of COPD-related inpatient hospitalizations (RR = 0.96 [0.79, 1.16], *p* = 0.66); COPD-related ED visits (RR = 1.11 [0.97, 1.28], *p* = 0.13); and OCS/antibiotics filled within 10 days of a COPD outpatient/office visit (RR = 1.01 [0.94, 1.09], *p* = 0.72).

**Table 2:** **COPD exacerbation rates and pneumonia events during the post-index period**

	**Adjusted rate** ^**1**^		**Rate ratio**	**95% CI**		**P-value** ^**1**^
	**BFC (n = 3,697)**	**FSC (n = 3,697)**		**Lower**	**Upper**	
Primary Outcome: COPD exacerbation rate	0.88	0.86	1.02	0.96	1.09	0.5637
COPD-related inpatient hospitalization	0.06	0.07	0.96	0.79	1.16	0.6644
COPD-related ED visit	0.14	0.13	1.11	0.97	1.28	0.1304
OCS and/or antibiotics filled within 10 days after a COPD-related outpatient/office visit	0.67	0.66	1.01	0.94	1.09	0.7153
Sensitivity analysis of primary outcome						
Stopping follow-up at ICS/LABA switch^2^ (within first 12 months of follow-up)	0.87	0.86	1.01	0.95	1.08	0.6787
Using all follow-up^3^ (≥12 months follow-up)	0.86	0.86	1.01	0.95	1.07	0.7678
Severe COPD exacerbation^4^ (12 month post-index period)	0.21	0.20	1.03	0.92	1.16	0.5760
	**% with ≥1 event**		**Odds Ratio**	**95% CI**		
	**BFC (n = 3,697)**	**FSC (n = 3,697)**		**Lower**	**Upper**	**P-value** ^**1**^
Pneumonia events: any event	17.3	19.0	0.92	0.81	1.04	0.1926
Inpatient hospitalization	8.9	10.3	0.87	0.75	1.02	0.0937
ED visit	1.0	1.3	0.80	0.51	1.23	0.3052
Outpatient/office visit	12.0	12.6	0.97	0.84	1.12	0.6404

^1^: Statistical comparisons are comparing BFC to FSC (reference group). Model covariates include sum of inpatient hospital stays >5 days (yes vs. no), LTRA use (0, 1, 2+), geographic region, peripheral vascular disease / atherosclerosis (yes vs. no), index prescribing physician specialty, and analogous pre-index variable (e.g., when analyzing the number of COPD related hospitalizations in the post-index, the model controlled for the number of pre-index COPD related hospitalizations).

^2^: Patients who filled an ICS/LABA that was different than the index medication during the 12 month post-index period had follow-up stopped on the date of the switch.

^3^: Using all follow-up: Patients were followed as long as possible until the end of the continuous health plan enrollment, or the end of the study period.

^4^: Severe COPD exacerbation includes only events due to COPD related inpatient hospitalization and COPD related ED visit.

CI: confidence interval; ED: emergency department.

### Sensitivity analyses of the primary outcome

As shown in Table 2, the sensitivity analyses were all consistent with the main analysis, showing no observed differences in exacerbation rates between the two treatment groups. Stopping follow-up at the presence of an ICS/LABA switch resulted in exacerbation rates for BFC and FSC of 0.87, and 0.86, respectively, and a RR of 1.01 [0.95, 1.08], *p* = 0.68. Using all follow-up time (median 2.1 years and maximum 4.1 years in each group) resulted in RR = 1.01 [0.95, 1.07], *p* = 0.77, and limiting to only severe COPD exacerbation events led to a similar finding (RR = 1.03 [0.92, 1.16], *p* = 0.58).

### Secondary outcomes

#### Time to first exacerbation

**Figure 2:**
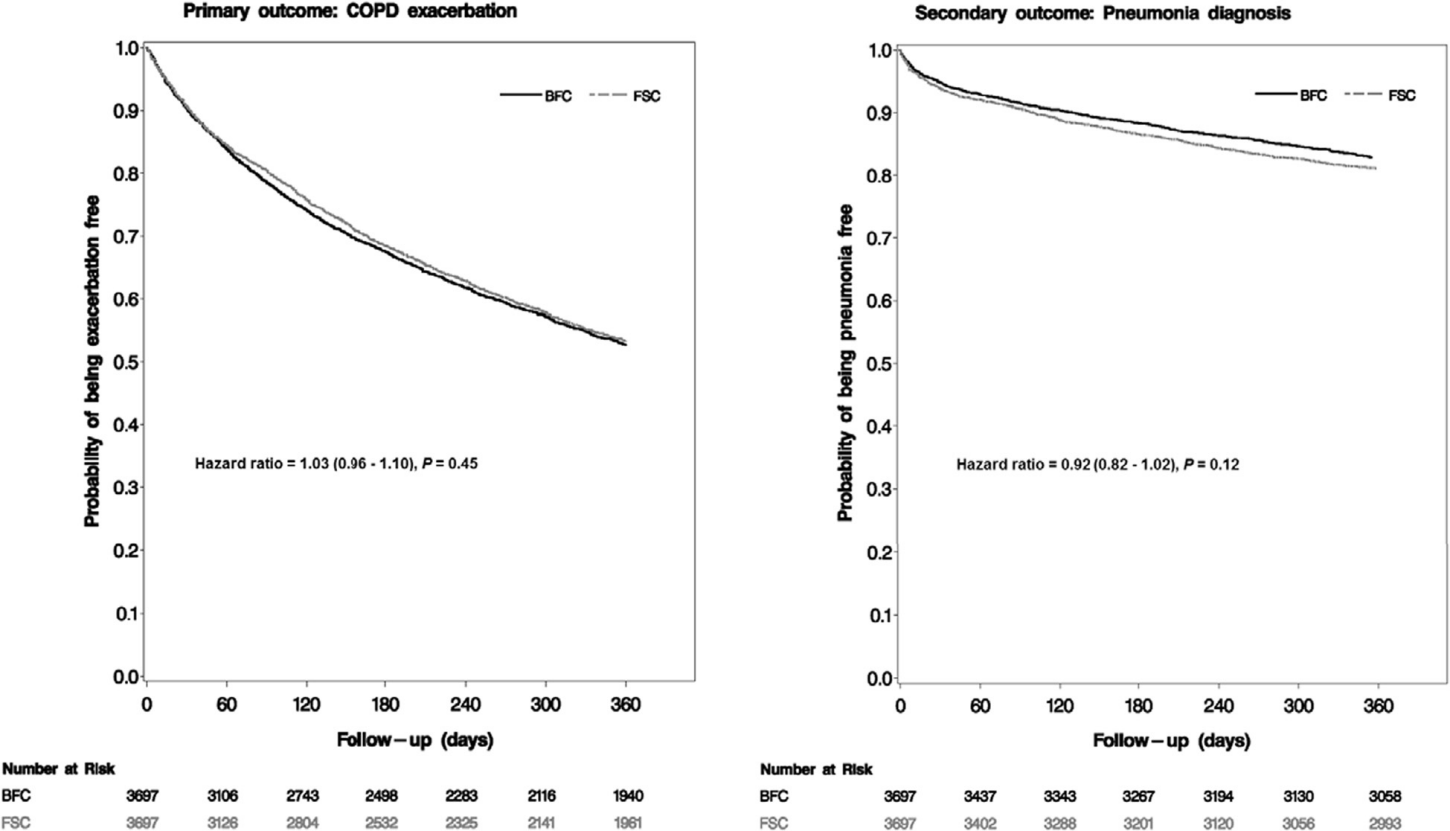
Kaplan-Meier curves for time to first COPD exacerbation and pneumonia diagnosis.

The analysis of time to first exacerbation was consistent with that of the exacerbation rates (Hazard ratio [HR] = 1.03 [0.96, 1.10], *p* = 0.45), in that there were no differences in risk of COPD exacerbation for patients initiating BFC compared with those initiating FSC, as shown in Figure 2.

### Medication use and healthcare utilization

#### Respiratory medication use

Respiratory medication use was similar for both groups. Approximately a third of patients received LAMA therapy post-index and nearly half had at least one fill of SABA medication, with no difference observed between the two groups. OCS was also received by more than 40% of the patients, while antibiotics were used by nearly three-quarters of the patients in each group, as shown in Table 3.

**Table 3:** **COPD medication use – adherence to index medication and prescription fills for other respiratory medication classes during the post-index period**

						**95% CI**		
	**BFC (n = 3,697)**		**FSC (n = 3,697)**		**Estimate** ^**1**^	**Lower**	**Upper**	**P-value**
Adherence to index medication								
Number of fills for index medication (including index fill) (mean, SD)	3.8	3.2	4.0	3.4	−0.20	−0.34	−0.05	0.0071
Patients with only the index fill (n, %)	1,252	33.9%	1208	32.7%	0.95	0.87	1.03	0.2197
Patients with 2 fills (n, %)	563	15.2%	575	15.6%				
Patients with 3 fills (n, %)	393	10.6%	398	10.8%				
Patients with 4 or more fills (n, %)	1,489	40.3%	1516	41.0%				
Proportion of days covered with index medication (mean, SD)	0.33	0.28	0.34	0.29	−0.01	−0.02	0.00	0.0914
COPD respiratory medications (number of patients with at least one fill)								
ICS monotherapy use (n, %)	220	6.0%	230	6.2%	0.94	0.78	1.14	0.5250
LAMA monotherapy use (n, %)	1,188	32.1%	1,226	33.2%	0.95	0.86	1.05	0.3232
LABA monotherapy use (n, %)	87	2.4%	78	2.1%	1.12	0.82	1.53	0.4807
Roflumilast (n, %)	23	0.6%	18	0.5%	1.23	0.66	2.29	0.5052
Theophylline use (n, %)	123	3.3%	111	3.0%	1.09	0.84	1.42	0.5120
SABA (n, %)	1,815	49.1%	1,845	49.9%	0.96	0.87	1.05	0.3550
SAMA (n, %)	169	4.6%	178	4.8%	0.95	0.77	1.18	0.6591
SABA/SAMA combination use (n, %)	656	17.7%	646	17.5%	1.03	0.91	1.16	0.6465
OCS monotherapy use (n, %)	1,628	44.0%	1,544	41.8%	1.09	0.99	1.20	0.0674
LTRA monotherapy use (n, %)	498	13.5%	429	11.6%	1.03	0.86	1.25	0.7316
Omalizumab use (n, %)	7	0.2%	5	0.1%	1.30	0.41	4.10	0.6556
Antibiotics use (n, %)	2,686	72.7%	2,667	72.1%	1.02	0.92	1.13	0.7239

^1^: Covariate adjusted mean differences are used for adherence measures from negative binomial (number of fills) and normal linear regression (proportion of days covered) models. The covariate adjusted odds ratio from an ordinal logistic regression is reported for the overall distribution of number of index medication fills (1, 2, 3, or 4+). Covariate adjusted odds ratios from logistic regression models are reported for the proportions of patients with at least one fill for each medication class. BFC is the reference group for all comparisons. Model covariates include: Sum of inpatient hospital stays >5 days (0 vs. 1), LTRA use (0, 1, 2+), geographic region, Peripheral vascular disease / atherosclerosis (0 vs. 1), index prescribing physician specialty, and analogous pre-index variable (for analysis of COPD medication classes only).

#### Adherence to the index medication

Patients in the BFC cohort filled their index medication 3.8 times on average during the 12-month follow-up period compared with 4.0 times for FSC patients (mean difference = −0.20, [−0.34, −0.05], p = 0.01), and 33.9% of BFC patients filled only their index medication without a refill in the next 12 months, compared with 32.7% of FSC patients. This resulted in a low average PDC in both groups (BFC: mean = 0.33 ± 0.28; FSC: mean = 0.34 ± 0.29), signaling that just one-third of the follow-up period was covered by supply of either medication on average.

#### Healthcare resource utilization

Use of healthcare resources was similar between the two cohorts during the 12-month post-index period. Just 6.2% of BFC patients and 6.9% of FSC patients had at least one COPD-related hospitalization (odds ratio (OR) = 0.91 [0.76, 1.10]), with an average length of stay of 7 days. COPD-related ED visits were present in 12% of patients from each group (OR = 1.08 [0.93, 1.24]), and BFC patients with at least one COPD-related outpatient visit had 9.6 visits on average compared with 10.0 in the FSC group (adjusted mean difference = −0.26 [−0.63, 0.14]). There remained no difference between the two groups when examining all-cause resource utilization (Table 4).

**Table 4:** **Healthcare resource utilization during the 12 month post-index period**

							Estimate*	**95% CI**		**P-value**
	**BFC (N = 3,697)**			**FSC (N = 3,697)**				**Lower**	**Upper**	
**COPD-related healthcare resource utilization**										
**COPD-related inpatient hospitalizations**										
Number of patients with ≥1 event (n, %)	228	6.2%		255	6.9%		0.91	0.76	1.10	0.3466
Number of events (mean, SD, median)^1^	1.3	1.1	1.0	1.2	0.7	1.0	0.10	−0.08	0.32	0.3016
Length of stay per patient (mean, SD, median)^1^	7.0	9.1	4.0	7.2	10.5	4.0	0.04	−0.96	1.21	0.9485
**COPD-related ICU stays**										
Number of patients with ≥1 event (n, %)	24	0.6%		32	0.9%		0.73	0.43	1.25	0.2564
Number of events (mean, SD, median)^1^	1.3	0.7	1.0	1.1	0.3	1.0	0.13	−0.33	0.85	0.6391
Length of stay per patient (mean, SD, median)^1^	1.8	2.0	1.0	2.7	8.5	1.0	0.16	−0.66	1.73	0.7661
**COPD-related ED visits**										
Number of patients with ≥1 event (n, %)	453	12.3%		438	11.8%		1.08	0.93	1.24	0.3123
Number of events (mean, SD, median)^1^	1.4	0.9	1.0	1.4	0.9	1.0	0.02	−0.11	0.18	0.7462
**COPD-related outpatient/office visits**										
Number of patients with ≥1 event (n, %)	3,047	82.4%		3,072	83.1%		0.93	0.82	1.05	0.2500
Number of events (mean, SD, median)^1^	9.6	11.9	5.0	10.0	12.8	5.0	−0.26	−0.63	0.14	0.1966
**All-cause healthcare resource utilization**										
**All-cause inpatient hospitalizations**										
Number of patients with ≥1 event (n, %)	1,131	30.6%		1,178	31.9%		0.98	0.89	1.09	0.7422
Number of events (mean, SD, median)^1^	1.9	1.5	1.0	2.0	1.7	1.0	0.04	−0.06	0.14	0.4678
Length of stay per patient (mean, SD, median)^1^	9.3	14.6	5.0	11.2	17.2	5.0	−0.10	−0.44	0.25	0.5528
**All-cause ICU stays**										
Number of patients with ≥1 event (n, %)	220	6.0%		241	6.5%		0.95	0.78	1.15	0.5631
Number of events (mean, SD, median)^1^	1.2	0.6	1.0	1.3	0.7	1.0	0.00	−0.18	0.21	0.9906
Length of stay per patient (mean, SD, median)^1^	2.0	5.5	1.0	1.9	4.2	1.0	0.00	−0.22	0.26	0.9910
**All-cause ED visits**										
Number of patients with ≥1 event (n, %)	1,012	27.4%		1,003	27.1%		1.05	0.94	1.16	0.4070
Number of events (mean, SD, median)^1^	1.6	1.4	1.0	1.7	1.7	1.0	0.02	−0.08	0.14	0.6778
**All-cause outpatient/office visits**										
Number of patients with ≥1 event (n, %)	3,679	99.5%		3,669	99.2%		1.59	0.88	2.88	0.1274
Number of events (mean, SD, median)^1^	33.1	30.7	25.0	34.9	32.3	27.0	0.03	−0.44	0.52	0.8925

*: Odds ratio from logistic regression is used for dichotomous variables (0 vs.1); Odds ratio from ordinal logistic regression is used for ordinal variables (0 vs. 1 vs. 2+); mean difference from negative binomial models is used for count variables. Statistical comparisons are comparing BFC to FSC (reference group); i.e., Mean diff = mean (BFC)-mean(FSC) and OR = Odds(BFC)/Odds(FSC). Model covariates include: Sum of inpatient hospital stays >5 days (0 vs. 1), LTRA use (0, 1, 2+), geographic region, Peripheral vascular disease / atherosclerosis (0 vs. 1), index prescribing physician specialty, and analogous pre-index variable.

^1^: Including only patients with at least one event; length of stay defined as the number of days from admission to discharge. Same date admission and discharge were counted as one.

### Pneumonia analysis

#### Post-index pneumonia diagnosis and utilization

The proportion of patients diagnosed with pneumonia during the 12 months following the initiation of therapy was similar for patients in each group (BFC = 17.3%, FSC = 19.0%, OR = 0.92 [0.81, 1.04], *p* = 0.19), as shown in Table 2. When examined by place of service, there remained no difference in pneumonia-related events between groups. Pneumonia-related inpatient hospitalizations occurred in 8.9% of BFC patients vs. 10.3% of the FSC group (OR = 0.87 [0.75, 1.02], *p* = 0.09), pneumonia-related ED visits were 1.0% vs. 1.3% (OR = 0.80 [0.51, 1.23], *p* = 0.31), pneumonia-related outpatient/office visits were 12.0% vs. 12.6% (OR = 0.97 [0.84, 1.12], *p* = 0.64), and the time to first pneumonia event was similar (HR = 0.92 [0.83, 1.02], *p* = 0.12), as shown in Figure 2.

## Discussion

In this study of matched cohorts of COPD patients initiating ICS/LABA treatment, no differences were observed in the effectiveness of BFC and FSC for all the outcome measures evaluated. No difference was observed between the two groups on the primary outcome measure, COPD exacerbation rates, which was defined by inpatient hospitalizations, emergency department visits, and antibiotic/OCS medication use paired with a COPD outpatient visit. Furthermore, the comparability of the two treatments remained consistent for all the sensitivity analyses and secondary measures as well.

Pneumonia, which is an important safety endpoint for COPD patients initiating ICS/LABA treatment, [5,15,16] was observed for a similar proportion of patients in the BFC and FSC cohorts during the follow up period. A Cochrane systematic review reported that budesonide and fluticasone, by themselves or with LABA combination treatment, were associated with increased pneumonia events but were not significantly associated with mortality. The review noted that comparisons of the treatments showed no significant difference in serious pneumonia, deaths or adverse events [15]. However, prior research has suggested a lower rate of pneumonia outcomes in those treated with BFC rather than FSC, [14] and a study of ICS therapies showed decreased rates of pneumonia in budesonide patients indirectly compared with those treated with fluticasone, [24] and similar results were reported in a meta-analysis of randomized clinical trials [25]. The lack of difference in our study may be due to low adherence rates when compared with the series of clinical trials, [25] which showed relatively low rates of discontinuation, and the Swedish observational study by Janson et al. [14] which analyzed only patients who were currently treated with drug. There may have also been differences in disease severity between the study populations, but we did not have access to clinical measurements to appropriately categorize COPD severity. However, the method of selecting eligible patients for the current study was similar to that in the Janson study, in that the only requirement was a diagnosis code for COPD prior to initiation of therapy and there was no requirement of having a prior exacerbation or any other clinical event. Although the baseline periods are different between our study (1 year) and the Swedish study (2 years), comparable proportions of patents with a prior COPD exacerbation (77% in Janson et al. vs. 62% in our study) and a prior pneumonia diagnosis (25% vs. 23%) were observed.

While the findings of this study indicate similar effectiveness of BFC and FSC with respect to COPD measures, this is in contrast to findings from a number of earlier studies that examined these two drugs. A Canadian study that followed a smaller matched sample (N = 2262) for a similar duration as our study, reported that COPD patients receiving BFC were less likely to be hospitalized or use ED services and required less anticholinergic medications than patients on FSC. The authors cautioned, however, that the observational nature of their study precluded any conclusions that medication alone was responsible for the observed effects [12].

A study conducted with data from the Swedish national registry system (21,361 medical records) over a much longer follow-up period (11 years) reported lower rates across all exacerbation outcomes among patients receiving BFC relative to FSC for moderate-to-severe COPD [13]. In contrast to our study, the Swedish registry study had a longer treatment duration (3.5 years on average) and only assessed outcomes for patients while they were on treatment. The present analysis was performed using an intent-to-treat design where outcomes may be influenced by poor adherence to treatment. Patients who remain on treatment for longer periods of time might experience different outcomes from what was observed in the present study. This could also indicate that one year of observation following treatment initiation may not be enough time to assess effectiveness between these two medications, and that relative effects between BFC and FSC manifest much later in treatment.

A study conducted by Roberts et al. in the US found that BFC and FSC had comparable effectiveness in terms of COPD exacerbation rates and pneumonia events, although the BFC patients required less rescue medication during the observation period. Similar to the current study, this study had a relatively short follow-up period, with only up to 6 months post treatment initiation which may not have been long enough to observe differences in COPD exacerbations and pneumonia events [11].

In general, the exacerbation rates found in the present study are smaller than some other studies, likely due to the fact that COPD severity is milder in this population that did not require prior exacerbations for inclusion, and did not have spirometry data to confirm diagnoses. It should also be noted that there are device differences for BFC in various countries (e.g., Turbuhaler in Sweden versus pressurized metered-dose inhaler in the US); however we do not believe that device type would be a primary reason for observed differences between the studies.

Despite differences between the findings of our study and those from earlier research, the findings of this study will be a valuable addition to this area because it drew from treatment data linked to a sizeable, commercially-insured population across the entire country facilitating access to a large number of patients with geographic diversity. The retrospective nature of the study enabled both looking backwards and forwards from a given point in time (the index date) without having to actively follow patients over time as is required in a prospective study.

More than 30% of patients in each group had a diagnosis of asthma during the pre-index period. Such patients are traditionally excluded from COPD studies, including clinical trials. By including potential asthma-COPD overlap patients in this study the findings have much greater generalizability to the COPD population.

The study used a robust study design in which patients were propensity-score matched (resulting in well balanced treatment groups across pre-index characteristics) and followed for an entire year. The Random Forest method utilized in the propensity score algorithm has been shown to be more effective in balancing treatment cohorts with less bias compared with traditional logistic regression techniques [21]. Another important strength was that pneumonia diagnoses were validated via medical record abstraction, which found an 80% positive predictive value of pneumonia diagnoses in the claims data when compared with medical records.

### Limitations

The results of this study must be viewed against several limitations, although none are expected to have differentially affected the treatment groups. The results are only generalizable to a commercially insured US population, and are based on data which are primarily collected for billing and reimbursement purpose, and may be subject to potential coding errors and inconsistencies. This study was conducted within a real-world setting and patients were not required to continuously take the index medication during follow-up. Individuals filled their index medication an average of approximately 4 times, which resulted in them having medication for 33%-34% of the follow-up period on average, thus this study compares patients initiating BFC therapy versus FSC therapy, but not necessarily patients who were actively taking medication. In fact, 34% of BFC patients and 33% of FSC patients only had a single fill of the index medication. Because of this, and because patients were taking other respiratory medications during the follow-up period (for example more than 30% of patients were also taking LAMA therapy), we cannot attribute outcomes solely to the initiated ICS/LABA therapy.

There was a lack of detailed clinical information, including smoking status and pulmonary function assessment, to adjust for COPD disease severity and activity because such information is absent from the primary data source, administrative claims. However, the study matched on prior COPD exacerbations which are the best predictor of future exacerbations [5]. The follow-up time in this study might have been insufficient to evaluate a less frequent event (i.e., pneumonia). Exacerbation rates could be underestimated as we did not capture events through OCS or antibiotic prescription dispensing via telephone (in the absence of an office visit) and most inpatient administered medications are not present in the claims data. Off-label use of the study medications (e.g., FSC 500/50 μg) was not captured, and there might be an ICS dose relationship to the increased risk of pneumonia which could not be examined here. Lastly, this was a non-randomized study, which may detect associations but causation cannot be inferred.

## Conclusion

No differences were found between patients initiating BFC or FSC in a real-world setting, in terms of rates of exacerbations, measured by COPD-related inpatient hospitalizations, emergency department visits, and outpatient visits with antibiotic/OCS medication use. This result was consistent across all sensitivity analyses and secondary endpoints. Pneumonia rates during the first year of initiating the therapy were also similar between the two groups, with similar rates of healthcare resource utilization and respiratory medication use.

## Abbreviations

BFC: Budesonide/formoterol combination
COPD: Chronic obstructive pulmonary disease
ED: Emergency department
FSC: Fluticasone/salmeterol combination
GOLD: Global initiative for chronic obstructive lung disease
HIPAA: Health insurance portability and accountability act
HIRE: HealthCore integrated research environment
IRB: Investigational review board
ICD-9-CM: International classification of diseases, clinical modification, 9th revision
ICS: Inhaled corticosteroid
LABA: Long-acting β_2_-agonist
LAMA: Long-acting muscarinic antagonist
LTRA: Leukotriene receptor antagonist
OCS: Oral corticosteroid
PDC: Proportion of days covered
SABA: Short acting β2-agonist
SAMA: Short-acting muscarinic antagonist
